# An Initiator-Free Electrochemical Approach to Radical Thiol–Ene Coupling in a Microfluidic Reactor

**DOI:** 10.3390/molecules31030429

**Published:** 2026-01-26

**Authors:** Kakeru Yamamoto, Kenta Arai

**Affiliations:** 1 Department of Chemistry, School of Science, Tokai University, Kitakaname, Hiratsuka-shi 259-1292, Kanagawa, Japan; 2 Institute of Advanced Biosciences, Tokai University, Kitakaname, Hiratsuka-shi 259-1292, Kanagawa, Japan

**Keywords:** thiol, thioether, electrochemistry, flow chemistry, microreactor, sustainable chemistry

## Abstract

The anti-Markovnikov addition of thiyl radicals, generated via one-electron oxidation of thiols, to C=C double bonds is a useful method for synthesizing unsymmetrical sulfides and has been widely applied in the preparation of pharmaceuticals and functional materials. However, conventional radical thiol–ene reactions require metal-based photoinitiators or organic photosensitizers, raising concerns about product isolation and environmental impact. Herein, we demonstrate an initiator-free thiol–ene coupling via electrochemical oxidation of thiols. Using a microfluidic electrochemical reactor, the electrochemically generated thiyl radicals undergo rapid and selective addition to alkenes, affording thioethers in reasonable yields. Substrate scope studies involving 13 alkenes and 13 thiols indicate that thiol acidity (p*K*_a_), alkene electronic properties, and steric effects play key roles in determining reaction efficiency. Although further optimization is required to improve yields and broaden substrate scope, this electrochemical approach highlights the potential of thiol–ene coupling as a sustainable tool in green synthetic chemistry.

## 1. Introduction

**Scheme 1:**
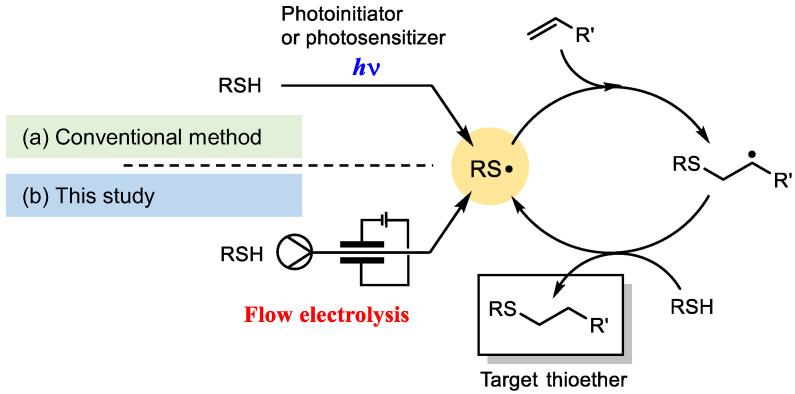
Photochemically (**a**) and electrochemically (**b**) initiated radical-mediated thiol–ene coupling.

Cross-coupling techniques with high regio- and chemoselectivity have significantly advanced the efficiency of pharmaceutical and materials development while contributing to the reduction in environmental impact. The reaction of thiols with C=C double bonds (enes) under appropriate conditions enables the efficient formation of thioether bonds and has been widely applied as one of the representative cross-coupling strategies [1,2,3]. The thiol–ene coupling reaction traces its origin to the Michael-type hydrothiolation of C=C double bonds with thiols, first reported by Posner in 1905 [4]. Meanwhile, the anti-Markovnikov addition of thiyl radicals, generated via one-electron oxidation of thiols, to electron-rich or electron-deficient C=C double bonds has also been recognized as one of the most general methods for thioether synthesis (Scheme 1) [1].

This radical-mediated thiol–ene reaction is classified as a type of “click reaction [5]” due to its high chemoselectivity, minimal byproduct formation, and ability to rapidly afford products in high yields. As a result, this reaction has found widespread application not only as a fundamental organic transformation but also as a versatile chemical modification tool in diverse fields, including materials science, drug discovery, and peptide and protein science [6,7,8,9,10,11,12,13,14,15,16,17,18]. The generation of thiyl radicals via single-electron oxidation of thiols is typically achieved under ultraviolet, visible, or near-infrared light irradiation in the presence of radical initiators, organic photosensitizers, or metal-based and metal-free photocatalysts [19,20,21,22,23,24,25,26,27,28,29,30,31,32,33] (path **a** in Scheme 1). However, these additives not only complicate purification after the reaction but also raise concerns regarding cost and biological toxicity. Therefore, in the context of green chemistry, further development of efficient and sustainable radical thiol–ene reactions remains highly desirable.

In an electrochemical reactor, the anode and cathode serve as the oxidant and reductant, respectively, enabling redox reactions to proceed without chemical redox reagents [34,35]. By taking advantage of this feature, it should be possible to generate thiyl radicals under initiator-free conditions and thereby promote the thiol–ene reaction (path **b** in Scheme 1). Only a limited number of studies have reported the electrochemical single-electron oxidation of thiols to generate thiyl radicals [36], as well as their subsequent addition to alkene substrates [37]. However, these transformations have not been systematically investigated as a synthetic methodology in organic chemistry. This approach is expected to simplify the reaction system, reduce environmental impact, and facilitate product purification, potentially providing a promising alternative to photochemical radical initiation methods.

**Figure 1:**
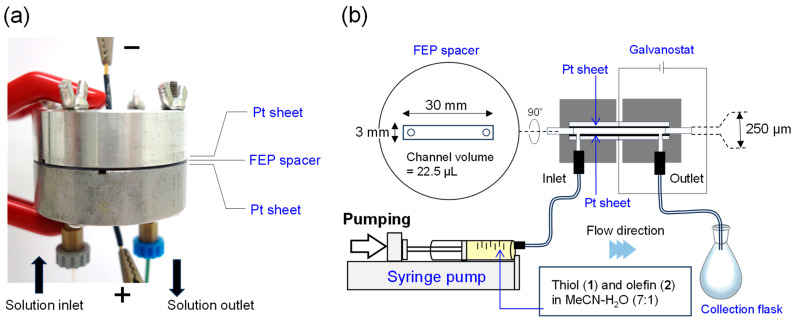
Electrochemical microreactor (ECMR). (**a**) Photograph of the ECMR used in this study. (**b**) General flow setup for the radical-mediated thiol–ene coupling reaction using the ECMR. The ECMR was independently constructed following the design reported by Wirth and co-workers [49]. For more information on the structure of the ECMR, see Supplementary Information (Figure S1).

Electrochemical microreactors (ECMRs), which combine the features of electrochemical and microflow reactors, have attracted considerable attention as next-generation tools for precision synthesis over the past few decades [38,39,40,41]. ECMRs are equipped with microflow channels sandwiched between two electrodes, where extremely short diffusion distances enable high spatiotemporal productivity (Figure 1). In short, combining electrochemical redox control with the rapid mixing and efficient mass transfer of flow reactors offers advantages such as high chemoselectivity, short reaction times, and facile scalability, which are difficult to achieve under conventional batch conditions. Indeed, employing various types of electrochemical microflow reactors, numerous highly efficient transformations have been demonstrated by many groups, including ours (see recent reviews [42,43,44,45,46]).

Wirth and co-workers have reported that trifluoroacetic acid (CF_3_CO_2_H) and difluoroacetic acid (CF_2_HCO_2_H) undergo anodic Kolbe electrolysis in an ECMR analogous to that shown in Figure 1, generating the corresponding CF_3_• and CF_2_H• radicals. These radicals rapidly react with electron-deficient alkenes at room temperature to afford the corresponding trifluoromethylated and difluoromethylated derivatives in reasonable yields [47]. Similarly, our group independently demonstrated that application of the same type of ECMR system enables the addition of electrochemically generated CF_3_• and CF_2_H• radicals to thiol and disulfide substrates, affording the corresponding CF_3_- and CF_2_H-substituted thioethers [48]. Together, these results suggest that ECMRs are well suited for efficient radical generation via anodic single-electron oxidation and for promoting their sequential and selective addition to diverse substrates.

In this study, we demonstrate an electrochemical radical thiol–ene coupling reaction based on the anodic single-electron oxidation of thiols, without the use of photocatalysts, photosensitizers, or radical initiators, as a new application of ECMRs, and show that ECMRs can serve as effective reaction devices for this class of transformations.

## 2. Results and Discussion

### 2.1. Optimization of Reaction Conditions

To investigate whether ECMR technology enables electrochemical radical thiol–ene coupling, a model reaction was conducted using methyl 3-mercaptopropionate (**1a**) and acrylamide (**2a**) (Table 1). A mixture of **1a** (0.1 mol L^−1^) and **2a** (0.1 mol L^−1^) in MeCN/H_2_O (7:1, *v*/*v*) was electrolyzed under a constant current (8.3 mA cm^−2^) in a continuous flow system using an ECMR (Figure 1) equipped with platinum anode and cathode electrodes and a FEP flow channel (22.5 µL) (Table 1, entry 1). Although both **1a** and **2a** were consumed within a residence time of 4.5 min, the desired thioether **3a** was not obtained; instead, disulfide **4** and its overoxidized product **5** were formed as byproducts in 16% and 12% yields, respectively.

To address this issue, the addition of Et_3_N (10 mol% with respect to **1a**) as an additive was examined (Table 1, entry 2). Under these conditions, the desired thioether was obtained in 22% isolated yield without formation of side products **4** and **5**, suggesting that deprotonation of the thiol plays a key role in the electrochemical single-electron oxidation leading to thiyl radical generation. Notably, no thiol–ene coupling via non-electrolytic Michael-type addition was observed when the reaction solution was left at room temperature for 24 h in the syringe, indicating that the radical thiol–ene coupling occurred exclusively within the ECMR under electrochemical conditions. Further increasing the amount of Et_3_N slightly decreased the yield and led to the formation of byproducts **4** and **5** (Table 1, entry 3). Next, the reaction was conducted under a lower constant current (4.2 mA cm^−2^) (Table 1, entry 4). Although complete consumption of the starting materials was observed after a longer residence time (9.0 min), no significant improvement in yield was achieved. In contrast, electrolysis under a higher constant current (16.7 mA cm^−2^) enabled complete conversion of the starting materials within a shorter residence time (2.3 min), but resulted in the formation of byproducts **4** and **5** and a decreased isolated yield of the desired product (Table 1, entry 5). Finally, the relationship between substrate concentration and yield was investigated. When the charge amount of either **1a** or **2a** was increased (Table 1, entries 6–8), the yield improved, reaching up to 69% (based on the amount of **2a** used) when the ratio of **1a** to **2a** was 2:1.

To demonstrate the advantage of the flow method, a batch reaction was performed using a mixed solution of **1a** (0.2 M), **2a** (0.1 M), and Et_3_N (0.1 M) in MeCN/H_2_O (7:1, 10 mL) with two platinum plate electrodes in an undivided cell under constant current (8.3 mA cm^−2^) (Table 1, entry 9). Complete conversion required 3 h, affording **3a** in 18% isolated yield along with disulfide **4** and its overoxidized byproduct **5**. These results highlight the superior selectivity, rate, and yield of the flow system over the batch method for electrochemical radical thiol–ene coupling.

**Figure 2:**
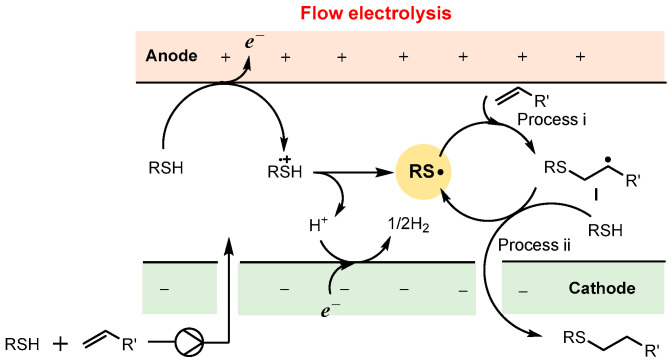
Plausible reaction mechanism for the electrochemical thiol–ene coupling under flow conditions, initiated by anodic single-electron oxidation of the thiol.

The proposed reaction mechanism occurring inside the reactor is summarized in Figure 2. Briefly, the thiol substrate initially undergoes anodic single-electron oxidation to form the corresponding radical cation, which subsequently undergoes deprotonation to generate the key intermediate, a thiyl radical. The thiyl radical rapidly adds to the olefin to form the radical intermediate **I** (process i). Subsequent radical transfer between intermediate **I** and another molecule of the thiol substrate affords the desired thioether product (process ii). Consistent with this proposed thiyl-radical chain mechanism, the thiol–ene coupling was significantly inhibited by the addition of 2,6-di-tert-butyl-4-methylphenol (BHT; a radical scavenger, 1.0 equiv relative to the thiol), resulting in a decrease in yield from 69% to 42%, which supports the involvement of radical intermediates in the reaction pathway. Hydrogen gas is generated at the cathode via proton reduction and is visible at the reactor outlet; however, the amounts produced do not interfere with the reaction.

Owing to the advantages of flow operation, the product formed at the electrode interface is immediately transported out of the reactor, thereby minimizing overoxidation and enabling selective formation of the thioether. Because the reaction is conducted in a two-electrode flow system without a reference electrode, the applied cell voltage represents an operational parameter reflecting the overall electrochemical driving force rather than the intrinsic thiol oxidation potential. Accordingly, the applied voltages were selected empirically to maintain efficient thiol oxidation while suppressing overoxidation, with the optimized conditions affording low and stable cell voltages (ca. 4.1–4.3 V, Table 1). Within this practical voltage window, selective thiol oxidation was achieved without significant solvent and olefin decomposition.

### 2.2. Thiol Substrate Scope

To explore the substrate scope of the electrochemical thiol–ene coupling, the conditions from entry 6 in Table 1 were applied to various thiol substrates (Table 2). For evaluation of thiol scope, acrylamide (**2a**) was used as the coupling partner. First, the applicability of aliphatic thiols was examined. When Boc-protected 2-aminoethanethiol (**1b**) was used as the thiol substrate, the corresponding sulfide **3b** was obtained in 70% isolated yield without byproduct formation. In contrast, when a cysteine derivative (**1c**) was used as the substrate, the amino acid derivative **3c** was obtained in 31% yield. The lower yield may be partly attributed to steric effects of the substituents on **1c** during coupling between the thiyl radical and **2a**. In addition, the use of cyclohexanethiol (**1d**) as a simple aliphatic thiol afforded only a trace amount of the corresponding thioether **3d**. This low yield is presumably due to the high p*K*_a_ of the thiol group, which may have impeded the deprotonation step required for electrochemical generation of the thiyl radical (Figure 2). However, in the case of 1-hexanethiol (**1e**), immediate non-electrolytic thiol–ene Michael addition occurred upon mixing with the olefin substrate under basic conditions, thereby preventing accurate evaluation of the electrochemical process.

Next, the applicability of aromatic thiols was evaluated. When benzenethiol (**1f**) was used, the corresponding sulfide (**3f**) was obtained in a modest yield (37%). In contrast, *para*-fluoro substitution improved the yield of sulfide **3g** to 57%, possibly due to the inductive effect of the fluorine atom, which lowers the thiol’s p*K*_a_ and facilitates generation of the thiyl radical. Although *para*-chloro substitution should also, in principle, increase the thiol’s acidity relative to benzenethiol (**1f**), the *para*-chloro thiol (**1h**) gave a lower yield of sulfide **3h** than **3f**, contrary to expectation. By contrast, *p*-bromobenzenethiol (**1i**) was insoluble in the solvent system and could not be evaluated. Moreover, although *para*-nitro substitution, an electron-withdrawing group, was expected to effectively afford the corresponding thioether, multiple complex byproducts were generated, probably due to undesired redox processes involving the nitro group of the thiol substrate (**1j**), and the desired thioether (**3j**) was obtained in only 9% yield. Additionally, *para*-methyl- and *para*-methoxy-substituted benzenethiols (**1k** and **1l**), bearing electron-donating groups, afforded **3k** (32%) and **3l** (11%), respectively, in lower yields than **3f**. Meanwhile, when benzylthiol was used, multiple unidentified byproducts were formed, and the desired product (**3m**) was not obtained.

### 2.3. Olefin Substrate Scope

Next, the substrate scope of alkenes was investigated under the optimized conditions (Table 1, entry 6) using **1a** as the coupling thiol (Table 3). When methyl acrylate (**2b**) was employed, symmetric sulfide **3n** was obtained in 54% yield. Ethyl acrylate (**2c**) and *tert*-butyl acrylate (**2d**) gave the corresponding sulfides **3o** (48%) and **3p** (43%), respectively, in slightly lower yields, likely due to increased C=C electron density from the more electron-donating ester groups, which reduces reactivity toward the thiyl radical. Presumably for the same reason, when methyl pent-4-enoate (**2e**) was used as the substrate, the corresponding sulfide **3q** was obtained only in trace amounts. Although thiyl radicals are generally described as electrophilic and are often expected to add preferentially to electron-rich alkenes, the present results indicate that alkene reactivity under the electrochemical flow conditions is not governed solely by the initial radical addition step (Figure 2, process i). Instead, the overall efficiency of the thiol–ene process may be influenced by subsequent radical chain propagation steps (Figure 2, process ii) as well as competing electrochemical pathways. In particular, relatively electron-rich alkenes are more susceptible to direct anodic oxidation and other side reactions near the electrode surface, which can suppress productive thiol–ene coupling. Consistent with this view, when styrene (**2f**) was employed as the substrate, a complex mixture of products was formed, reflecting the susceptibility of electron-rich alkenes to competing anodic oxidation and radical side reactions under the applied electrochemical conditions.

Methyl methacrylate (**2g**) and methyl crotonate (**2h**), with β- or α-methyl substitution, provided **3s** (43%) and **3t** (24%), respectively. In contrast, when dehydroalanine derivative **2i** was used, the desired amino acid derivative **3u** was detected but could not be isolated, possibly because the two substituents at the α-carbon sterically hindered the addition and/or subsequent radical transfer (Figure 2, processes i and/or ii).

Furthermore, when cyclic alkenes such as cyclopentene (**2j**), norbornene (**2k**), maleic anhydride (**2l**), and *N*-cyclohexylmaleimide (**2m**) were used as substrates, the corresponding thioethers were obtained only in trace amounts or not at all. These results suggest that alkene reactivity in the present system is influenced by a combination of electronic effects, structural constraints imposed by cyclic frameworks, and the efficiency of radical chain propagation under electrochemical conditions. In particular, strongly electron-deficient cyclic alkenes such as maleic anhydride and maleimide derivatives may be disfavored both in the initial thiyl radical addition and in subsequent chain-propagation steps due to the altered reactivity of the post-addition carbon-centered radical (**I**).

## 3. Materials and Methods

### 3.1. General

The electrochemical microreactor (ECMR), similar to that developed by Wirth [49], was independently constructed. Melting points were measured using a Yanako MP-S3 micro melting-point system (Yanaco Group, Kyoto, Japan) and are uncorrected. ^1^H (500 MHz) and ^13^C (125.8 MHz) NMR spectra were recorded on a Bruker AV-500 spectrometer (Bruker Japan, Yokohama, Japan) at 298 K. Coupling constants (*J*) are reported in Hz. High-resolution mass spectra (HRMS) were obtained using a JMS-T100LP AccuTOF LC-Express instrument (JEOL Ltd., Tokyo, Japan) under atmospheric-pressure chemical ionization (APCI) conditions. All reactions were monitored by thin-layer chromatography (TLC), which was performed on pre-coated plates of silica gel 60 (Merck, Minato-ku, Japan). The substrates **1b** [50], **1c** [51], and **2i** [51] were prepared according to reported procedures. All other chemicals were purchased from commercial suppliers and used without further purification.

### 3.2. General Procedure for Electrochemical Thiol–Ene Coupling Using ECMR

Thiol **1** (1.20 mmol), olefin **2** (0.60 mmol), and Et_3_N (0.12 mmol, 16.8 μL) were dissolved in MeCN/H_2_O (7:1, *v*/*v*; 6.0 mL), and a 5 mL portion of the resulting solution was introduced into the ECMR equipped with an FEP spacer containing a flow channel (0.3 cm × 3.0 cm × 250 μm) using a syringe pump (flow rate: 5.0 μL min^−1^; residence time: 4.5 min) under a constant current of 15 mA (current density: 8.3 mA cm^−2^) and collected in a round-bottom flask at the outlet. The solution was concentrated to 1 mL under reduced pressure. The resulting solution was added with H_2_O (20 mL) and extracted with EtOAc (3 × 20 mL). The combined organic layers were washed with saturated aqueous NH_4_Cl solution (1 × 25 mL) and then with brine (1 × 25 mL). The organic phase was passed through a column-type phase separator (ISOLUTE^®^, Biotage Japan, Koto-ku, Japan) to remove any residual aqueous layer and then concentrated under reduced pressure. The crude residue was purified by silica gel column chromatography (EtOAc/*n*-hexane).

### 3.3. Electrochemical Thiol–Ene Coupling of ***1a*** with ***2a*** Under Batch Conditions

A mixture of **1a** (2.00 mmol, 240.4 mg), **2a** (1.00 mmol, 72.7 mg), and Et_3_N (0.20 mmol, 27.9 μL) dissolved in MeCN/H_2_O (7:1, *v*/*v*; 10.0 mL) was stirred and electrolyzed under aerobic conditions at 25 °C in an undivided cell (39.5 mm tall and 26.6 mm in diameter) equipped with two platinum foils (1.5 cm × 1.0 cm) as the anode and cathode. A constant current density (8.3 mA cm^−2^) was applied. After TLC analysis (silica gel, EtOAc/*n*-hexane, *v*/*v* = 2:1) confirmed complete consumption of the starting materials, the electrolysis was stopped. The resulting mixture was concentrated to 2 mL under reduced pressure and diluted with H_2_O (20 mL). The aqueous phase was extracted with EtOAc (6 × 20 mL). The combined organic layers were washed with saturated aqueous NH_4_Cl solution (1 × 50 mL) and then with brine (1 × 50 mL). The organic phase was passed through a column-type phase separator (ISOLUTE^®^, Biotage Japan, Koto-ku, Japan) to remove any residual aqueous layer and then concentrated under reduced pressure. The crude residue was purified by silica gel column chromatography (EtOAc/*n*-hexane, 2:1, *v*/*v*). Fractions with *R*_f_ = 0.40 (EtOAc/*n*-hexane, 2:1, *v*/*v*) on TLC were combined and concentrated to give **3a** as a white solid (35.7 mg, 18%).

## 4. Conclusions

In this study, we developed a radical thiol–ene coupling reaction driven by the electrochemical single-electron oxidation of thiols without the use of radical initiators. Conducting the reaction in an electrochemical microreactor (ECMR) enabled rapid and selective addition of the generated thiyl radicals to alkenes in an anti-Markovnikov manner, affording the corresponding thioethers with minimal formation of disulfides or overoxidized byproducts. Substrate scope studies indicated that the yields were mainly governed by thiol acidity, the electronic nature of the alkene, and steric effects, with more acidic thiols and electron-deficient alkenes often showing enhanced reactivity under the optimized conditions. However, several deviations from these trends were also observed, indicating that reaction efficiency is governed by multiple factors beyond simple thiol p*K*_a_ and electronic considerations of olefins. These include the reactivity of the post-addition carbon-centered radical involved in chain propagation, as well as competing electrochemical decomposition pathways of starting materials, intermediates, or products. Although a broad range of thiols were compatible with the reaction, the generality toward alkenes was relatively limited and warrants further study. The current solvent system is restricted to MeCN/H_2_O mixtures, which constrains the substrate scope due to solubility issues. Moreover, improving reaction efficiency will likely require optimization of the electrode materials in accordance with the redox potentials of the thiols. Despite these limitations, this electrochemical strategy offers a sustainable cross-coupling platform that operates without metal catalysts, photosensitizers, or excess oxidants. Ongoing efforts are directed toward refining the reaction system and broadening its substrate scope to enable applications in the synthesis of pharmaceutical intermediates and functional materials.

## Figures and Tables

**Table 1:** Optimization of reaction conditions for electrochemical radical thiol–ene coupling.

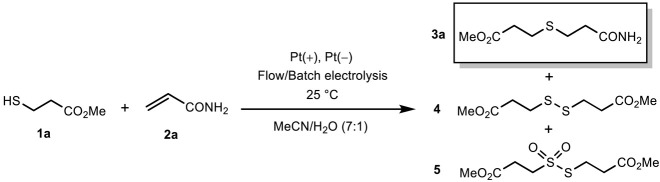										
**Entry**	**Flow/Batch**	**Concentration (mol L^−1^)**		**Additive**	**Reaction Time (Min) *^a^***	**Current Density** **(mA cm^−2^) *^b^***	**Cell Voltage** **(V) *^c^***	**Isolated Yield (%)**		
		**1a**	**2a**					**3a**	**4**	**5**
1	Flow	0.1	0.1	None	4.5	8.3	4.7–4.8	NG *^d^*	16	12
2		0.1	0.1	Et_3_N (10% mol)	4.5	8.3	4.6–5.0	22	NG	NG
3		0.1	0.1	Et_3_N (20% mol)	4.5	8.3	5.9–6.9	13	2	4
4		0.1	0.1	Et_3_N (10% mol)	9.0	4.2	4.0–4.5	19	NG	NG
5		0.1	0.1	Et_3_N (10% mol)	2.3	16.7	5.4–5.6	17	2	2
6		0.2	0.1	Et_3_N (10% mol)	4.5	8.3	4.1–4.2	69	NG	NG
7		0.3	0.1	Et_3_N (10% mol)	4.5	8.3	4.1–4.3	65	NG	NG
8		0.1	0.2	Et_3_N (10% mol)	4.5	8.3	6.5–7.2	16	NG	NG
9	Batch *^e^*	0.2	0.1	Et_3_N (10% mol)	180	8.3	8.3–23.1	18	trace *^f^*	7

*^a^* Under flow conditions, the reaction time is defined as the residence time, calculated as channel volume (22.5 μL)/flow rate (μL min^−1^). *^b^* Current densities were estimated without correction for the roughness of the electrode surfaces. *^c^* Cell voltage was recorded as the potential difference between the anode and cathode under constant-current conditions; values are reported as the range observed during the reaction. *^d^* Not generated. *^e^* The reaction was performed in an undivided cell (see Materials and Methods for experimental details). *^f^* Products were detected by APCI–MS analysis of the crude mixture but were not isolated.

**Table 2:** Electrochemical radical thiol-ene coupling using various thiol substrates *^a^*.

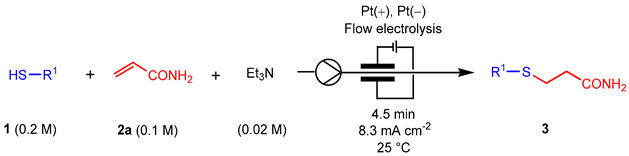			
**Thiol 1**		**Product 3**	**Isolated Yield (%)**
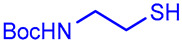	**1b**	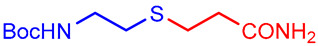	**3b**: 70%
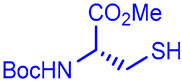	**1c**	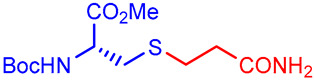	**3c**: 31%
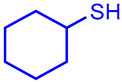	**1d**	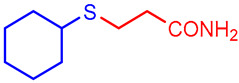	**3d**: trace *^b^*
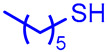	**1e**	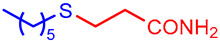	**3e**: 67% *^c^*
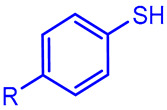	**1f** (X = H) **1g** (X = F) **1h** (X = Cl) **1i** (X = Br) **1j** (X = NO_2_) **1k** (X = Me) **1l** (X = OMe)	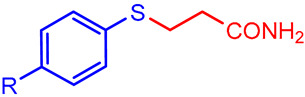	**3f** (X = H): 37% **3g** (X = F): 57% **3h** (X = Cl): 26% **3i** (X = Br): NR *^d^* **3j** (X = NO_2_): 9% **3k** (X = Me): 32% **3l** (X = OMe): 11%
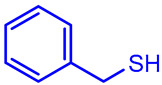	**1m**	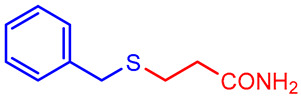	**3m**: NG *^e^*

*^a^* Reactions were conducted under the optimized conditions shown in entry 6 of Table 1. *^b^* Products were detected by APCI–MS analysis of the crude mixture but were not isolated. *^c^* A non-electrolytic thiol–ene Michael addition proceeded, preventing evaluation of the electrochemical reaction efficiency. *^d^* No reaction observed due to substrate insolubility. *^e^* Not generated.

**Table 3:** Electrochemical radical thiol–ene coupling using various olefin substrates *^a^*.

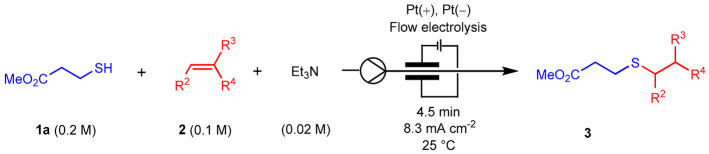			
**Olefine 2**		**Product 3**	**Isolated Yield (%)**
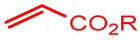	**2b** (R = Me) **2c** (R = Et) **2d** (R = tBu)	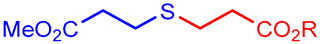	**3n** (X = Me): 54% **3o** (X = Et): 48% **3p** (X = tBu): 43%
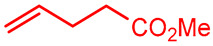	**2e**	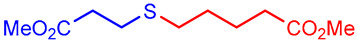	**3q**: trace *^b^*
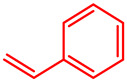	**2** **f**	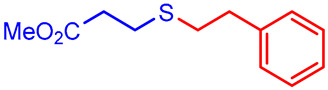	**3r**: NG
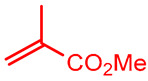	**2** **g**	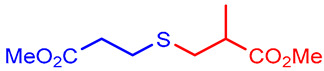	**3s**: 43%
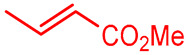	**2** **h**	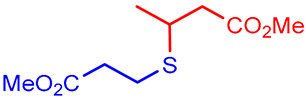	**3t**: 24%
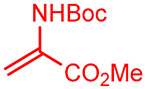	**2** **i**	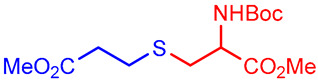	**3u**: trace
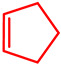	**2** **j**	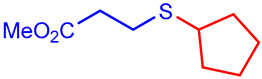	**3v**: NG *^c^*
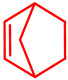	**2** **k**	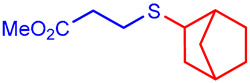	**3w**: trace
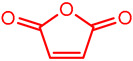	**2** **l**	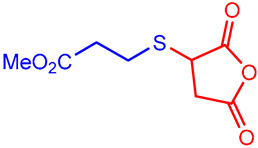	**3x**: trace
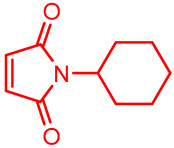	**2** **m**	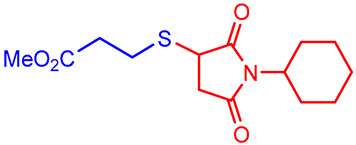	**3y**: trace

*^a^* Reactions were conducted under the optimized conditions shown in entry 6 of Table 1. *^b^* Products were detected by APCI–MS analysis of the crude mixture but were not isolated. *^c^* Not generated.

## Data Availability

The data supporting the findings of this study are available in the Supporting Information of this article.

## Supplementary Materials

The following supporting information can be downloaded at: https://www.mdpi.com/article/10.3390/molecules31030429/s1, Figure S1: Structure and setup of the electrochemical microreactor (ECMR); Experimental details and compound identification. The authors have cited additional references within the Supporting Information [52,53].

## Abbreviations

The following abbreviations are used in this manuscript:

APCI	Atmospheric Pressure Chemical Ionization
ECMR	Electrochemical microreactor
Et_3_N	Triethylamine
EtOAc	Ethyl acetate
FEP	Fluorinated ethylene propylene
LC-MS	Liquid Chromatography–Mass Spectrometry
MeCN	Acetonitrile
MgSO_4_	Magnesium sulfate
NMR	Nuclear Magnetic Resonance
TLC	Thin-Layer Chromatography
