# Supplementary material for: An Initiator-Free Electrochemical Approach to Radical Thiol–Ene Coupling in a Microfluidic Reactor
Source: Molecules. 2026 Jan 26;31(3):429. doi: 10.3390/molecules31030429 (PMC12898347; doi:10.3390/molecules31030429)
Supplement: Supplementary file 1 [file molecules-31-00429-s001.zip › molecules-4090636-supplementary.pdf]

*Supporting information*

# An Initiator-Free Electrochemical Approach to Radical Thiol–Ene Coupling in a Microfluidic Reactor

Kakeru Yamamoto[1] and Kenta Arai[1,2*]

[1.] Department of Chemistry, School of Science, Tokai University, Kitakaname, Hiratsuka-shi, Kanagawa 259-1292, Japan

[2.] Institute of Advanced Biosciences, Tokai University, Kitakaname, Hiratsuka-shi, Kanagawa 259-1292, Japan

---

## *Table of Contents*

**1. Supplemental Figure (Figure S1)** …2

**2. Experimental Details and Compound Identification.** …3

**3. References** …9

## 1. Supplemental figure

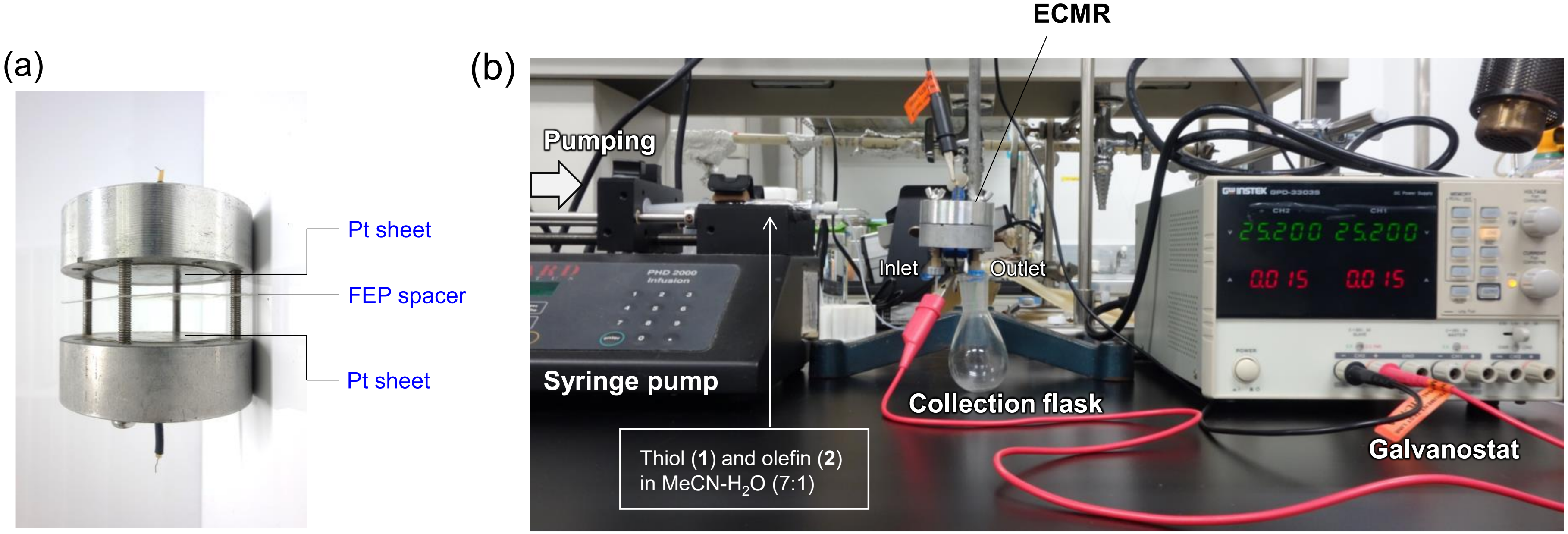


**Figure S1.** Structure and setup of the electrochemical microreactor (ECMR). (a) Exploded view of the device. (b) Photograph of the general microreactor setup.

## 2. Experimental Details and Compound Identification.

*The general experimental procedures are described in the main text (see Section 3, Materials and Methods).*

Methyl 3-((3-amino-3-oxopropyl)thio)propanoate

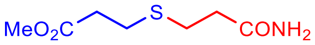

**3a**

The reaction was performed using **1a** (144.8 mg, 1.2 mmol), **2a** (43.0 mg, 0.6 mmol), and $Et_3N$ (16.8 µL, 0.12 mmol) in $MeCN/H_2O$ (7:1, v/v; 6.0 mL). A 5 mL portion of the reaction mixture was introduced into the ECMR for electrolysis. The crude product was purified by silica gel column chromatography (EtOAc/*n*-hexane, 2:1, v/v) to afford **3a** as a white solid.

Yield: 65.7 mg (69 %); $R_f$: 0.40 (EtOAc/*n*-hexane, 2:1, v/v); M.p: 43.5–44.6; $^1$H NMR (500 MHz, $CDCl_3$): δ = 3.70 (s, 3H), 3.37 (t, *J* = 7.0 Hz, 2H), 3.31 (t, *J* = 7.4 Hz, 2H), 2.88 (t, *J* = 7.7 Hz, 2H), 2.86 ppm (t, *J* = 7.0 Hz, 2H); $^{13}$C NMR (125.8 MHz, $CDCl_3$): δ = 170.8, 116.4, 52.7, 49.1, 48.8, 27.0, 11.3 ppm; HRMS (APCI-TOF) m/z: $[M–H]^-$ calcd for $C_7H_{12}NO_3S^-$, 190.0543 ; found, 190.0520.

*tert*-Butyl (2-((3-amino-3-oxopropyl)thio)ethyl)carbamate

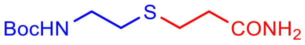

**3b**

The reaction was performed using **1b** (214.8 mg, 1.2 mmol), **2a** (43.0 mg, 0.6 mmol), and $Et_3N$ (16.8 µL, 0.12 mmol) in $MeCN/H_2O$ (7:1, v/v; 6.0 mL). A 5 mL portion of the reaction mixture was introduced into the ECMR for electrolysis. The crude product was purified by silica gel column chromatography (EtOAc) to afford **3b** as a yellow oil.

Yield: 86.4 mg (70 %); $R_f$: 0.77 (EtOAc); $^1$H NMR (500 MHz, $CDCl_3$): δ = 5.41 (t, *J* = 5.80 Hz, 1H), 3.67–3.59 (m, 2H), 3.40 (t, *J* = 7.3 Hz, 2H), 3.37–3.32 (m, 2H), 2.95 (t, *J* = 7.3 Hz, 1H), 1.45 ppm (s, 9H); $^{13}$C NMR (125.8 MHz, $CDCl_3$): δ = 155.9, 116.9, 80.3, 53.1, 48.6, 34.4, 28.3, 11.1 ppm; HRMS (APCI-TOF) m/z: $[M–H]^-$ calcd for $C_{10}H_{19}N_2O_3S^-$, 247.1122; found, 247.1131.

Methyl *S*-(3-amino-3-oxopropyl)-*N*-(tert-butoxycarbonyl)-L-cysteinate

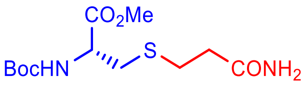
**3c**

The reaction was performed using **1c** (288.6 mg, 1.2 mmol), **2a** (43.0 mg, 0.6 mmol), and $Et_3N$ (16.8 µL, 0.12 mmol) in MeCN/$H_2O$ (7:1, v/v; 6.0 mL). A 5 mL portion of the reaction mixture was introduced into the ECMR for electrolysis. The crude product was purified by silica gel column chromatography (EtOAc/*n*-hexane, 2:1, v/v) to afford **3c** as a white solid.

Yield: 48.9 mg (31 %); $R_f$: 0.68 (EtOAc/*n*-hexane, 2:1, v/v); M.p: 134.8–137.2; $^1$H NMR (500 MHz, $CDCl_3$): δ = 5.66 (br d, $J$ = 5.2 Hz, 1H), 4.67 (br q, $J$ = 5.4, Hz, 1H), 3.83 (s, 3H), 3.81–3.73 (m, 2H), 3.40 (t, $J$ = 7.7 Hz, 2H), 2.91 (t, $J$ = 7.7 Hz, 2H), 1.47 ppm (s, 9H); $^{13}$C NMR (125.8 MHz, $CDCl_3$): δ = 169.5, 155.3, 116.3, 81.4, 54.6, 53.4, 50.1, 50.0, 28.2, 11.1 ppm; HRMS (APCI-TOF) m/z: $[M–H]^–$ calcd for $C_{12}H_{21}N_2O_5S^–$, 305.1177; found, 305.1148.

3-(Hexylthio)propanamide

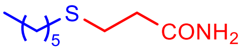
**3e**

The reaction was performed using **1e** (168.9 µL, 1.2 mmol), **2a** (43.0 mg, 0.6 mmol), and $Et_3N$ (16.8 µL, 0.12 mmol) in MeCN/$H_2O$ (7:1, v/v; 6.0 mL). In this reaction, a non-electrolytic Michael-type thiol–ene addition occurred spontaneously in the syringe. The crude product was purified by silica gel column chromatography (EtOAc/*n*-hexane, 2:1, v/v) to afford **3e** as a white solid.

Yield: 76.1 mg (67 %); $R_f$: 0.28 (EtOAc/*n*-hexane, 2:1, v/v); M.p.: 85.2–86.9; $^1$H NMR (500 MHz, $CDCl_3$): δ = 2.74 (t, $J$ = 7.3 Hz, 2H), 2.49–2.43 (m, 3H), 1.55–1.49 (m, 2H), 1.33–1.27 (m, 2H), 1.24–1.78 (m, 4H), 0.82 ppm (t, $J$ = 6.8 Hz, 3H); $^{13}$C NMR (125.8 MHz, $CDCl_3$): δ = 173.8, 36.0, 32.4, 31.4, 29.5, 28.5, 27.5, 22.5, 14.0 ppm; HRMS (APCI-TOF) m/z: $[M–H]^–$ calcd for $C_9H_{18}NOS^–$, 188.1115; found, 188.1136.

3-(Phenylthio)propanamide

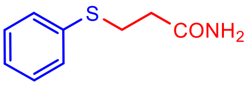
**3f**

The reaction was performed using **1f** (122.4 µL, 1.2 mmol), **2a** (43.0 mg, 0.6 mmol), and $Et_3N$ (16.8 µL, 0.12 mmol) in MeCN/$H_2O$ (7:1, v/v; 6.0 mL). A 5 mL portion of the reaction mixture was introduced into the ECMR for electrolysis. The crude product was purified by silica gel column chromatography (EtOAc/*n*-hexane, 2:1, v/v) to afford **3f** as a white solid.

Yield: 36.0 mg (37 %); $R_f$: 0.60 (EtOAc/*n*-hexane, 2:1, v/v); M.p.: 92.5–93.4; $^1$H NMR (500

MHz, $CDCl_3$): δ = 7.96–7.94 (m, 2H), 7.77–7.73 (m, 1H), 7.66–7.63 (m, 2H), 3.41 (t, $J$ = 7.4 Hz, 2H), 2.84 ppm (t, $J$ = 7.8 Hz, 2H); $^{13}C$ NMR (125.8 MHz, $CDCl_3$): δ = 137.5, 134.8, 129.8, 128.3, 116.0, 51.1, 12.0 ppm; HRMS (APCI-TOF) m/z: $[M–H]^-$ calcd for $C_9H_{10}NOS^-$, 180.0489; found, 180.0500.

Spectroscopic data were consistent with literature values [52].

3-((4-Fluorophenyl)thio)propanamide

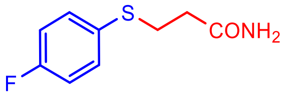


**3g**

The reaction was performed using **1g** (129.0 µL, 1.2 mmol), **2a** (43.0 mg, 0.6 mmol), and $Et_3N$ (16.8 µL, 0.12 mmol) in MeCN/$H_2O$ (7:1, v/v; 6.0 mL). A 5 mL portion of the reaction mixture was introduced into the ECMR for electrolysis. The crude product was purified by silica gel column chromatography (EtOAc/*n*-hexane, 2:1, v/v) to afford **3g** as a white crystal.

Yield: 56.7 mg (57 %); $R_f$: 0.63 (EtOAc/*n*-hexane, 2:1, v/v); M.p.: 120.1–121.0; $^1H$ NMR (500 MHz, $CDCl_3$): δ = 8.00–7.96 (m, 2H), 7.34–7.29 (m, 2H), 3.42 (t, $J$ = 7.4 Hz, 2H), 2.85 ppm (t, $J$ = 7.6 Hz, 2H); $^{13}C$ NMR (125.8 MHz, $CDCl_3$): δ = 166.4 (d, $J$ = 258.6 Hz), 133.7 (d, $J$ = 3.4 Hz), 131.3 (d, $J$ = 9.9 Hz), 117.2 (d, $J$ = 22.7 Hz), 116.0, 51.3, 12.0 ppm; HRMS (APCI-TOF) m/z: $[M–H]^-$ calcd for $C_9H_9FNOS^-$, 198.0394; found, 198.0395.

3-((4-Chlorophenyl)thio)propanamide

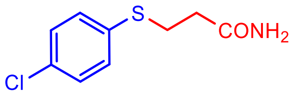


**3h**

The reaction was performed using **1h** (183.1 mg, 1.2 mmol), **2a** (43.0 mg, 0.6 mmol), and $Et_3N$ (16.8 µL, 0.12 mmol) in MeCN/$H_2O$ (7:1, v/v; 6.0 mL). A 5 mL portion of the reaction mixture was introduced into the ECMR for electrolysis. The crude product was purified by silica gel column chromatography (EtOAc/*n*-hexane, 2:1, v/v) to afford **3h** as a white solid.

Yield: 27.8 mg (26 %); $R_f$: 0.68 (EtOAc/*n*-hexane, 2:1, v/v); M.p.: 91.5–93.2; $^1H$ NMR (500 MHz, $CDCl_3$): δ = 7.91–7.88 (m, 2H), 7.64–7.61 (m, 2H), 3.41 (t, $J$ = 7.8 Hz, 2H), 2.86 ppm (t, $J$ = 7.6 Hz, 2H); $^{13}C$ NMR (125.8 MHz, $CDCl_3$): δ = 141.8, 136.0, 130.2, 129.8, 115.8, 51.2, 12.0 ppm; HRMS (APCI-TOF) m/z: $[M–H]^-$ calcd for $C_9H_9ClNOS^-$, 214.0099; found, 214.0100.

Spectroscopic data were consistent with literature values [52].

3-((4-Nitrophenyl)thio)propanamide

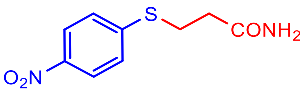

**3j**

The reaction was performed using **1j** (171.1 mg, 1.1 mmol), **2a** (39.6 mg, 0.55 mmol), and $Et_3N$ (15.2 µL, 0.11 mmol) in MeCN/$H_2O$ (7:1, v/v; 5.5 mL). A 5 mL portion of the reaction mixture was introduced into the ECMR for electrolysis. The crude product was purified by silica gel column chromatography (EtOAc/*n*-hexane, 2:1, v/v) to afford **3j** as a yellow solid.

Yield: 10.6 mg (9 %); $R_f$: 0.71 (EtOAc/*n*-hexane, 2:1, v/v); M.p.: 137.5–138.4; $^1$H NMR (500 MHz, $CDCl_3$): δ = 8.42–8.39 (m, 2H), 8.11–8.09 (m, 2H), 3.39 (t, *J* = 7.3 Hz, 2H), 2.83 ppm (t, *J* = 7.4 Hz, 2H); $^{13}$C NMR (125.8 MHz, $CDCl_3$): δ = 151.5, 143.2, 130.0, 125.0, 115.4, 51.2, 11.9 ppm; HRMS (APCI-TOF) m/z: $[M–H]^-$ calcd for $C_9H_9N_2O_3S^-$, 225.0339; found, 225.0342.

3-(p-Tolylthio)propanamide

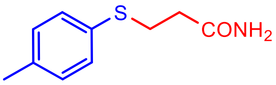

**3k**

The reaction was performed using **1k** (149.8 mg, 1.2 mmol), **2a** (43.0 mg, 0.6 mmol), and $Et_3N$ (16.8 µL, 0.12 mmol) in MeCN/$H_2O$ (7:1, v/v; 6.0 mL). A 5 mL portion of the reaction mixture was introduced into the ECMR for electrolysis. The crude product was purified by silica gel column chromatography (EtOAc/*n*-hexane, 2:1, v/v) to afford **3k** as a yellow solid.

Yield: 31.5 mg (32 %); $R_f$: 0.62 (EtOAc/*n*-hexane, 2:1, v/v); M.p.: 84.0–85.8; $^1$H NMR (500 MHz, $CDCl_3$): δ = 7.72 (d, *J* = 8.2 Hz, 2H), 7.33 (d, *J* = 8.3 Hz, 2H), 3.30 (t, *J* = 7.6 Hz, 2H), 2.73 (t, *J* = 7.6 Hz, 2H), 2.40 ppm (s, 3H); $^{13}$C NMR (125.8 MHz, $CDCl_3$): δ = 146.0, 134.5, 130.4, 128.3, 116.1, 51.2, 21.7, 12.1 ppm; HRMS (APCI-TOF) m/z: $[M–H]^-$ calcd for $C_{10}H_{12}NOS^-$, 194.0645; found, 194.0635.

Spectroscopic data were consistent with literature values [52].

3-((4-Methoxyphenyl)thio)propanamide

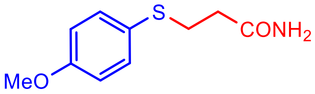

**3l**

The reaction was performed using **1l** (147.6 mg, 1.2 mmol), **2a** (43.0 mg, 0.6 mmol), and $Et_3N$ (16.8 µL, 0.12 mmol) in MeCN/$H_2O$ (7:1, v/v; 6.0 mL). A 5 mL portion of the reaction mixture

was introduced into the ECMR for electrolysis. The crude product was purified by silica gel column chromatography (EtOAc/*n*-hexane, 2:1, v/v) to afford **3l** as a yellow solid.
Yield: 12.0 mg (11 %); $R_f$: 0.63 (EtOAc/*n*-hexane, 2:1, v/v); M.p.: 81.2–83.0; $^1$H NMR (500 MHz, $CDCl_3$): δ = 7.91–7.88 (m, 2H), 7.01–6.98 (m, 2H), 3.83 (s, 3H), 3.29 (t, *J* = 7.5 Hz, 2H), 2.74 ppm (t, *J* = 7.8 Hz, 2H); $^{13}$C NMR (125.8 MHz, $CDCl_3$): δ = 164.6, 130.5, 128.8, 116.1, 115.0, 55.8, 51.4, 12.1 ppm; HRMS (APCI-TOF) m/z: $[M–H]^-$ calcd for $C_{10}H_{12}NO_2S^-$, 210.0594; found, 210.0603.

Dimethyl 3,3'-thiodipropionate

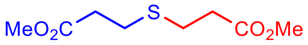


**3n**

The reaction was performed using **1a** (144.2 mg, 1.2 mmol), **2b** (53.8 µL, 0.6 mmol), and $Et_3N$ (16.8 µL, 0.12 mmol) in $MeCN/H_2O$ (7:1, v/v; 6.0 mL). A 5 mL portion of the reaction mixture was introduced into the ECMR for electrolysis. The crude product was purified by silica gel column chromatography (EtOAc/*n*-hexane, 2:1, v/v) to afford **3n** as a white solid.
Yield: 56.2 mg (54 %); $R_f$: 0.45 (EtOAc/*n*-hexane, 2:1, v/v); M.p.: 94.9–96.5; $^1$H NMR (500 MHz, $CDCl_3$): δ = 3.73 (s, 6H), 3.36 (t, *J* = 7.5 Hz, 4H), 2.87 ppm (t, *J* = 7.4 Hz, 4H); $^{13}$C NMR (125.8 MHz, $CDCl_3$): δ = 170.9, 52.5, 48.6, 26.7 ppm; HRMS (APCI-TOF) m/z: $[M–H]^-$ calcd for $C_8H_{13}O_4S^-$, 205.0540; found, 205.0549.
Spectroscopic data were consistent with literature values [53].

Ethyl 3-((3-methoxy-3-oxopropyl)thio)propanoate

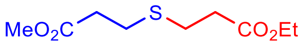


**3o**

The reaction was performed using **1a** (144.2 mg, 1.2 mmol), **2c** (65.4 µL, 0.6 mmol), and $Et_3N$ (16.8 µL, 0.12 mmol) in $MeCN/H_2O$ (7:1, v/v; 6.0 mL). A 5 mL portion of the reaction mixture was introduced into the ECMR for electrolysis. The crude product was purified by silica gel column chromatography (EtOAc/*n*-hexane, 2:1, v/v) to afford **3o** as a white solid.
Yield: 53.0 mg (48 %); $R_f$: 0.51 (EtOAc/*n*-hexane, 2:1, v/v); M.p.: 59.8–62.8; $^1$H NMR (500 MHz, $CDCl_3$): δ = 4.20 (q, *J* = 7.2 Hz, 2H), 3.75 (s, 3H), 3.37–3.34 (m, 4H), 2.91–2.87 (m, 4H), 1.28 ppm (t, *J* = 7.2 Hz, 3H); $^{13}$C NMR (125.8 MHz, $CDCl_3$): δ = 170.8, 170.3, 61.6, 52.5, 48.7, 48.6, 27.0, 26.8, 14.1 ppm; HRMS (APCI-TOF) m/z: $[M–H]^-$ calcd for $C_9H_{15}O_4S^-$, 219.0697; found, 219.0678.

*tert*-Butyl 3-((3-methoxy-3-oxopropyl)thio)propanoate

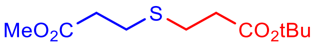

**3p**

The reaction was performed using **1a** (144.2 mg, 1.2 mmol), **2d** (87.4 µL, 0.6 mmol), and $Et_3N$ (16.8 µL, 0.12 mmol) in $MeCN/H_2O$ (7:1, v/v; 6.0 mL). A 5 mL portion of the reaction mixture was introduced into the ECMR for electrolysis. The crude product was purified by silica gel column chromatography (EtOAc/*n*-hexane, 2:1, v/v) to afford **3p** as a white solid.

Yield: 54.5 mg (43 %); $R_f$: 0.60 (EtOAc/*n*-hexane, 2:1, v/v); M.p.: 63.7–65.4; $^1$H NMR (500 MHz, $CDCl_3$): δ = 3.744 (s, 3H), 3.37–3.31 (m, 4H), 2.89 (t, *J* = 7.6 Hz, 2H), 2.80 (t, *J* = 7.5 Hz, 2H), 1.47 ppm (s, 9H); $^{13}$C NMR (125.8 MHz, $CDCl_3$): δ = 170.9, 169.5, 82.1, 52.5, 48.7, 48.5, 28.1, 28.0, 26.7 ppm; HRMS (APCI-TOF) m/z: $[M–H]^-$ calcd for $C_{11}H_{19}O_4S^-$, 247.1010; found, 247.1006.

Methyl 3-((3-methoxy-3-oxopropyl)thio)-2-methylpropanoate

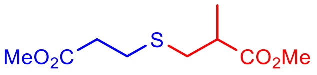

**3r**

The reaction was performed using **1a** (144.2 mg, 1.2 mmol), **2f** (64 µL, 0.6 mmol), and $Et_3N$ (16.8 µL, 0.12 mmol) in $MeCN/H_2O$ (7:1, v/v; 6.0 mL). A 5 mL portion of the reaction mixture was introduced into the ECMR for electrolysis. The crude product was purified by silica gel column chromatography (EtOAc/*n*-hexane, 2:1, v/v) to afford **3r** as a colorless oil.

Yield: 47.9 mg (43 %); $R_f$: 0.45 (EtOAc/*n*-hexane, 2:1, v/v); $^1$H NMR (500 MHz, $CDCl_3$): δ = 3.74 (s, 3H), 3.74 (s, 3H), 3.64 (dd, *J* = 7.8, 14.2 Hz, 1H), 3.36 (t, *J* = 7.5 Hz, 2H), 3.19–3.12 (m, 1H), 3.04 (dd, *J* = 5.2, 14.3 Hz, 1H), 2.88 (t, *J* = 7.6 Hz, 2H), 1.39 ppm (d, *J* = 7.3 Hz, 3H); $^{13}$C NMR (125.8 MHz, $CDCl_3$): δ = 174.2, 170.8, 55.7, 52.5, 52.4, 49.2, 34.2, 26.7, 17.7 ppm; HRMS (APCI-TOF) m/z: $[M–H]^-$ calcd for $C_9H_{15}O_4S^-$, 219.0697; found, 219.0721.

Methyl 3-((3-methoxy-3-oxopropyl)thio)butanoate

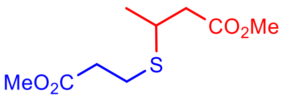

**3s**

The reaction was performed using **1a** (144.2 mg, 1.2 mmol), **2g** (63.2 µL, 0.6 mmol), and $Et_3N$ (16.8 µL, 0.12 mmol) in $MeCN/H_2O$ (7:1, v/v; 6.0 mL). A 5 mL portion of the reaction mixture was introduced into the ECMR for electrolysis. The crude product was purified by silica gel

column chromatography (EtOAc/*n*-hexane, 2:1, v/v) to afford **3s** as a yellow oil.

Yield: 27.2 mg (24 %); $R_f$: 0.62 (EtOAc/*n*-hexane, 2:1, v/v); $^1$H NMR (500 MHz, $CDCl_3$): δ = 3.75 (s, 3H), 3.74 (s, 3H), 3.60–3.54 (m, 1H), 3.33 (t, *J* = 7.5 Hz, 2H), 3.08 (dd, *J* = 4.5, 16.8 Hz, 1H), 2.89 (t, *J* = 7.7 Hz, 2H), 2.53 (dd, *J* = 9, 16.7 Hz, 1H), 1.47 ppm (d, *J* = 7.0 Hz, 3H); $^{13}$C NMR (125.8 MHz, $CDCl_3$): δ = 170.9, 170.6, 54.4, 52.5, 52.4, 45.4, 33.7, 26.2, 13.9 ppm; HRMS (APCI-TOF) m/z: $[M–H]^-$ calcd for $C_9H_{15}O_4S^-$, 219.0697; found, 219.0690.

## 3. References


52. Liu, Y.; Lai, Z.; Yang, P.; Xu, Y.; Zhang, W.; Liu, B.; Lu, M.; Chang, H.; Ding, T.; Xu, H. Thio-Michael Addition of α,β-Unsaturated Amides Catalyzed by Nmm-Based Ionic Liquids. *RSC Adv.* **2017**, *7*, 43104–43113, doi:10.1039/C7RA08956B.
53. Sasano, Y.; Kogure, N.; Nagasawa, S.; Kasabata, K.; Iwabuchi, Y. 2-Azaadamantane *N*-Oxyl (AZADO)/Cu Catalysis Enables Chemoselective Aerobic Oxidation of Alcohols Containing Electron-Rich Divalent Sulfur Functionalities. *Org. Lett.* **2018**, *20*, 6104–6107, doi:10.1021/acs.orglett.8b02528.
